# Contribution of tryptophan and its metabolites to transplant outcome: a mini-review

**DOI:** 10.3389/fimmu.2024.1395421

**Published:** 2024-10-15

**Authors:** Darío Donoso-Meneses, Cristina Padilla, María José Moya-Guzmán, Maria-Luisa Alegre, Karina Pino-Lagos

**Affiliations:** ^1^ Facultad de Medicina, Centro de Investigación e Innovación Biomédica, Universidad de los Andes, Santiago, Chile; ^2^ Department of Medicine, Section of Rheumatology, University of Chicago, Chicago, IL, United States

**Keywords:** transplantation, trypthophan metabolism, microbiota, immune regulation, tolerance

## Abstract

Long-term tolerance in the absence of immunosuppressive drugs is a major goal in the transplantation field, not yet attained. Recent research on the role of commensal microbiota in the control of immunity has opened new avenues for the search of novel clinical interventions. Indeed, products of intestinal metabolism generated by both host cells and the microbiota have been identified as modulators of the immune response. Among these, tryptophan (Trp) and its derivatives are being investigated to understand their impact on alloimmunity and their potential usefulness as therapeutic targets to improve allograft survival. Here, we reviewed the latest findings on the contribution of Trp metabolic pathways to transplant outcomes.

## Introduction

Allograft rejection is a current challenge that limits transplant success. Since the use of immunosuppressive drugs present negative side effects on patients, there is still need for new approaches to prevent rejection (1). Tryptophan (Trp) metabolism has been associated with the immune response that drives transplant rejection or acceptance; thus, its manipulation may help address this problem. Therefore, this review aims to describe the latest findings on the contribution of Trp metabolic pathways to transplant outcomes, including the participation of the microbiota and microbiota-produced metabolites.

## Tryptophan metabolic pathways

Tryptophan (Trp) is one of the nine essential amino acids present in most protein-based foods that can be metabolized by host cells and gut microbiota. Its metabolism is involved in several biological processes that affect the central nervous system (CNS) and immunity (2). Trp is absorbed (mainly) in the small intestine; in circulation, it is found bound to albumin, although a small fraction can be protein-free (3). Cells uptake Trp metabolites through system L amino acid carrier (SLC) superfamily, specifically SLC3/7, which is sensed by G-protein-coupled membrane receptors (GPCRs) expressed on host intestinal cells (4).

**Figure 1:**
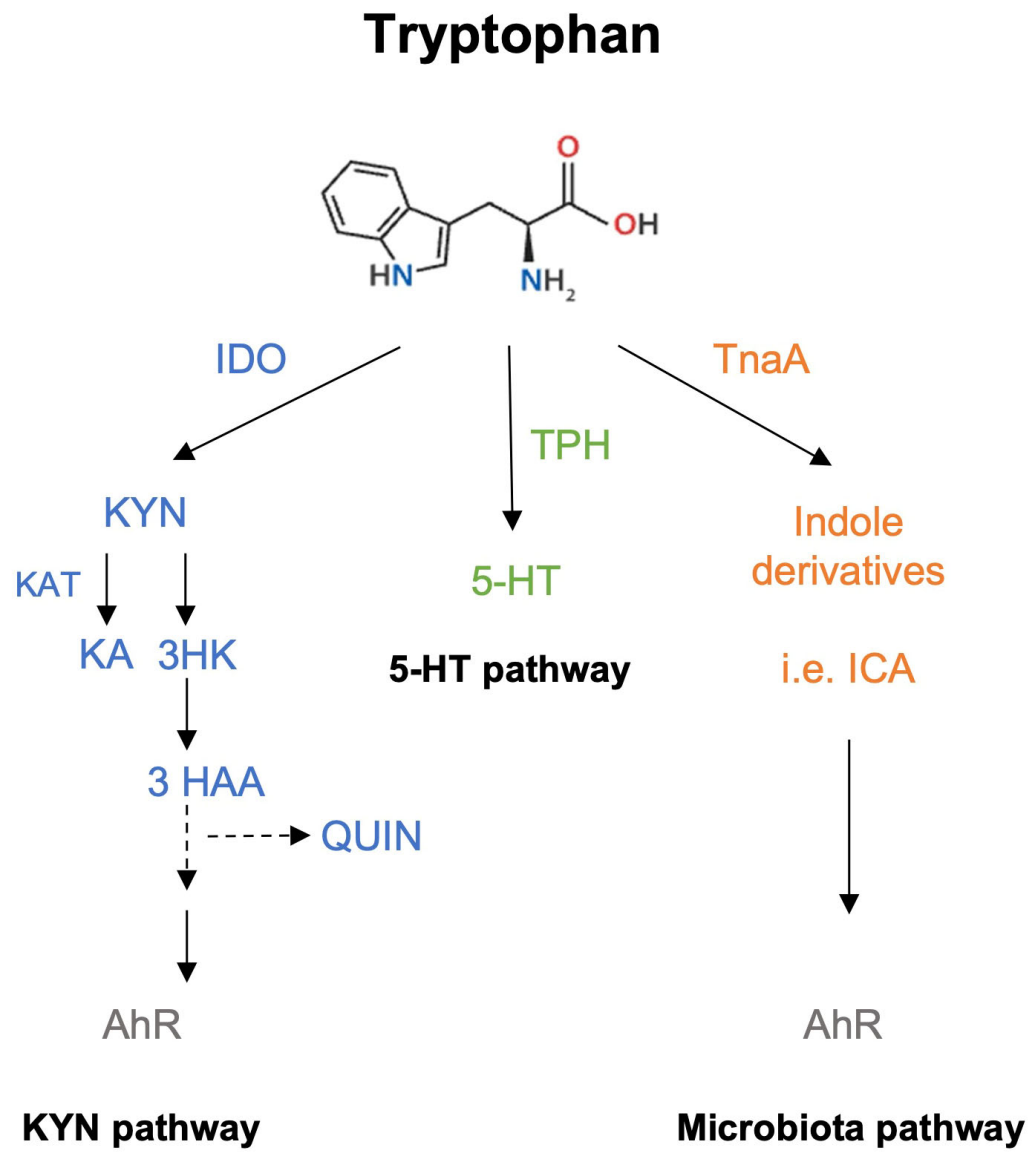
Schematic simplified representation of Trp catabolic pathways. Trp metabolism can take three different routes, the most active being the KYN pathway, which metabolizes ~90% of Trp. The first rate-limiting enzyme involved in this reaction is IDO, a protein expressed in hepatic and immune cells (macrophages and DCs). KYN, KA and QUIN are some of the final products obtained from this pathway and can bind to AhR. The second pathway involves the production of 5-HT through the activity of TPH, and the binding of 5-HT to 5-HT receptors or to serotonin receptors. The third pathway requires the microbiota, which can sense and metabolize Trp via tryptophanase (TnaA) activity to generate indole derivatives that also serve as AhR ligands.

Trp can be catabolized via three different pathways: the kynurenine (KYN) pathway, the serotonin (5-Hydroxytryptamine, 5-HT) pathway and the indole pathway (**Figure 1**). About 95% of Trp is used in the KYN pathway producing several kynurenines, such as KYN, kynurenic acid (KA), and quinolinic acid (QUIN) among others, whereas only 1-2% of Trp enters the 5-HT pathway in a reaction first catalyzed by Trp hydroxylase (TPH). Trp catabolism produces metabolites that can bind to N-methyl-D-aspartate (NMDA) receptors, explaining its role as a major neuromodulator (5). With respect to the KYN pathway, several reports have shown that KYN and QUIN exert negative (toxic) effects on cells; in contrast, KA is thought to have beneficial activity (5, 6). A major step in this pathway involves the 2 isoforms of indolamine 2,3-dioxygenase (IDO1/2), which catalyze the conversion of Trp to KYN and other metabolites in a rate-limiting manner (**Figure 1**). IDO1 is expressed on macrophages and dendritic cells (DCs) revealing the strong connection between Trp metabolism and the regulation of the immune response (6).

## Tryptophan metabolism in immune regulation: role of pivotal intermediaries

### Indolamine 2,3-dioxygenase

Most of the current studies about Trp metabolites and the immune system focus on two molecules: KYN and KA, which depend on IDO activity. IFN-γ induces IDO expression on APCs, and its activity results in T cell apoptosis (especially Th1) via Trp starvation and kynurenines toxicity, which results in Th2 skewing (7, 8). IDO activity also targets eosinophils, reducing their migration to inflamed sites (9). On plasmacytoid DC (pDC), Toll like Receptor 9 (TLR9) ligand stimulation by CpG results in IDO upregulation and Treg-mediated immune suppression (10). As expected, IDO deficiency results in loss of Treg function and FoxP3 expression, and acquisition of a Th17 phenotype (11).

### Kynurenine

As the main IDO product, KYN can induce FoxP3 expression on CD4+ T cells in an AhR-dependent manner (12). KYN can act directly on activated T cells via the SLC7A5 transporter, resulting in upregulation of AhR mRNA levels (13). Interestingly, the concentration of KYN is elevated in the plasma of cancer patients, which may induce a tolerogenic environment for tumor cells. Furthermore, inhibition of AhR signaling reduces immunosuppression, as demonstrated by lower frequencies of tumor-infiltrating Tregs and macrophages in tumor-bearing mice, leading to reduced tumor growth (14).

### Kynurenic acid

It has been described that KA binds to the G protein-coupled receptor 35 (GPR35) and can inhibit Tumor necrosis factor-alpha (TNF-α) secretion by lipopolysaccharide (LPS)-stimulated monocytes (15). GPR35 is expressed on several cells and tissues including immune and gastrointestinal cells in which KA induces the expression of anti-inflammatory genes, such as *Il4, Il13, Il33* and *Il10*, leading to diminished adiposity and weight loss in obese mice (16).

Using different animal models of gut inflammation (chemotherapy and colitis), intestinal damage was shown to increase KYN and KA production (17). In fact, KYN and KA triggered GPR35 and AhR pathways, and upregulated IDO and IL-6 upon cell damage. Tissue repair was then promoted through GPR35 negative feedback, inhibiting IL-6 production and, thus, controlling intestinal repair and homeostasis (17). TNF-stimulated gene-6 (TSG-6) is a secreted protein that plays a major role in tissue repair and has anti-inflammatory activity. Its functions are mediated through the interaction with several ligands such as chemokines, growth factors, enzymes (tryptases) and matrix proteins, and its expression is up regulated upon inflammation on mesenchymal stem cells (MSC) and immune cells (18). *In vitro*, TSG-6 prevents the expression of proinflammatory molecules such as induced Nitric oxide synthase (iNOS), IL-6 and TNF-α (19), and *in vivo*, TSG-6 controls inflammation, preventing skin fibrosis and favoring wound healing and tissue repair (20–22). Interestingly, it has been reported that KA can promote TSG-6 production through activation of AhR signaling on human MSC resulting in immunosuppression (23). Overall, IDO expression and the production of KYN and KA is activated upon inflammation, but their biological effects may regulate this response leading to immunosuppression and tissue repair, restoring homeostasis.

### Aryl hydrocarbon receptor

AhR is a ligand-dependent transcription factor. The use of agonists, antagonists, and cell targeted AhR deficiency in animals has shown AhR’s involvement in the pathogenesis of several diseases, including inflammatory disorders, endocrine perturbations, premature aging, and cancer (24). Recent evidence has dissected the importance of AhR signaling on intestinal homeostasis, where alteration of AhR and its immunomodulatory function reveals changes in the architecture of the intestine, such as homeostasis and its permeable barrier function (25). In line with these antecedents, a recent study showed that activation of the AhR pathway with its ligand 6-formylindolo [3,2-b] carbazole (FICZ) is required for controlling cell proliferation, inflammation and integrating dietary metabolites signals on intestinal epithelial cells (26).

Regarding immune cells, AhR signaling on mouse and human APCs diminished inflammation by inhibiting APC maturation and cytokine release, especially IL-6 (27, 28). In B cells, BCR activation in the presence of IL-4 triggered AhR expression, which is required for B cells to enter the cell cycle. In contrast, in B regulatory cells, AhR appears necessary for optimal production of IL-10, as AhR deficiency in this cell population drove autoimmunity and differentiation of pro-inflammatory B cells (29, 30). Also, it has been demonstrated that KYN is preferentially up taken by activated T cells via SLC receptors and that it can bind to AhR to regulate T cell metabolism (13). In fact, AhR signaling on CD4+ T cells lead to FoxP3 and IL-10 expression through the activation of the fatty acid oxidation pathway. This effect was observed *in vivo* in mouse models of colitis and arthritis, in which animals treated with an AhR agonist showed increased frequencies of Tregs whereas those treated with a short hairpin RNA targeting AhR had fewer Tregs (31). Furthermore, AhR positively controls T cell differentiation in a TGF-β-dependent manner driving *de novo* generation of Tregs. In contrast, TGF-β can also synergize with the high affinity AhR ligand FICZ to activate AhR, resulting in Th17 instead of Tregs differentiation (32). Another study demonstrated that dietary Trp deficiency triggered dysbiosis, depletion of kynurenines and other amino acid-related metabolites, leading to upregulation of RAR-related orphan receptor-γ+ (RORγt) Tregs over GATA binding protein 3+ (GATA3) Tregs, with RORγt+ Tregs being linked to bacterial infection. As expected, this effect is mediated by AhR signaling (33). Thus, AhR signaling fine tunes the Treg/Th17 balance based on the activation strength of the signaling pathway.

### Intestinal microbiota

The intestinal microbiota metabolizes ~5% of the Trp that is not absorbed by the intestine. This reaction is carried out by Gram-negative and Gram-positive bacteria through the activity of the tryptophanase A (TnaA) enzyme. This drives the formation of indole molecules, heterocyclic compounds with diverse activities, such as maintenance of bacterial communities, and immunoregulation of host intestinal health (34). Interestingly, KYN and derived indoles act as ligands for AhR (**Figure 1**). Indeed, germ-free (GF) mice or with a generalized dysbiosis do not produce those AhR ligands, suggesting a relevant role of gut microbiota in the activation of this pathway (35).

*In vitro* studies indicate that indoles have an anti-inflammatory effect because their addition to human enterocytes results in downregulation of IL-18 secretion and NF-κB activation, and stimulation of IL-10 production (36). In an *in vivo* model of colitis, administration of indoles changed the composition of intestinal DCs, limiting the presence of CD103-CD11b+ DCs which promote the secretion of IFN-γ and IL-17, suggesting that administration of indole acid derivatives might serve as an anti-inflammatory therapy (37). Furthermore, intestinal indoles can also activate AhR signaling pathways in gut tissue, promoting the secretion of IL-22, a cytokine that controls fungal infection and inflammation, in addition to the production of IL-10 which possesses intestinal anti-inflammatory properties (38, 39).

The regulation of immune cells by gut-microbiota metabolites is also observed in the context of oncological treatments (40). For example, the microbiota-derived Trp metabolite indole-3-acetic acid (3-IAA) was shown to be enriched in patients with pancreatic cancer that respond to chemotherapy. Besides, fecal transplantation and oral 3-IAA administration increased chemotherapy efficacy in humanized gnotobiotic murine models of pancreatic cancer (41). Notably, in other report the administration of indole-3-carboxaldehyde (3-IAld, a microbial Trp catabolite) prevented gastrointestinal toxicity associated to immune checkpoint inhibitors therapies, maintaining their therapeutic properties and efficacy against melanoma (42). These effects were related to a change on microbiota composition through the AhR/IL-22 axis and to be independent of IDO and IL-10 activity (42).

**Figure 2:**
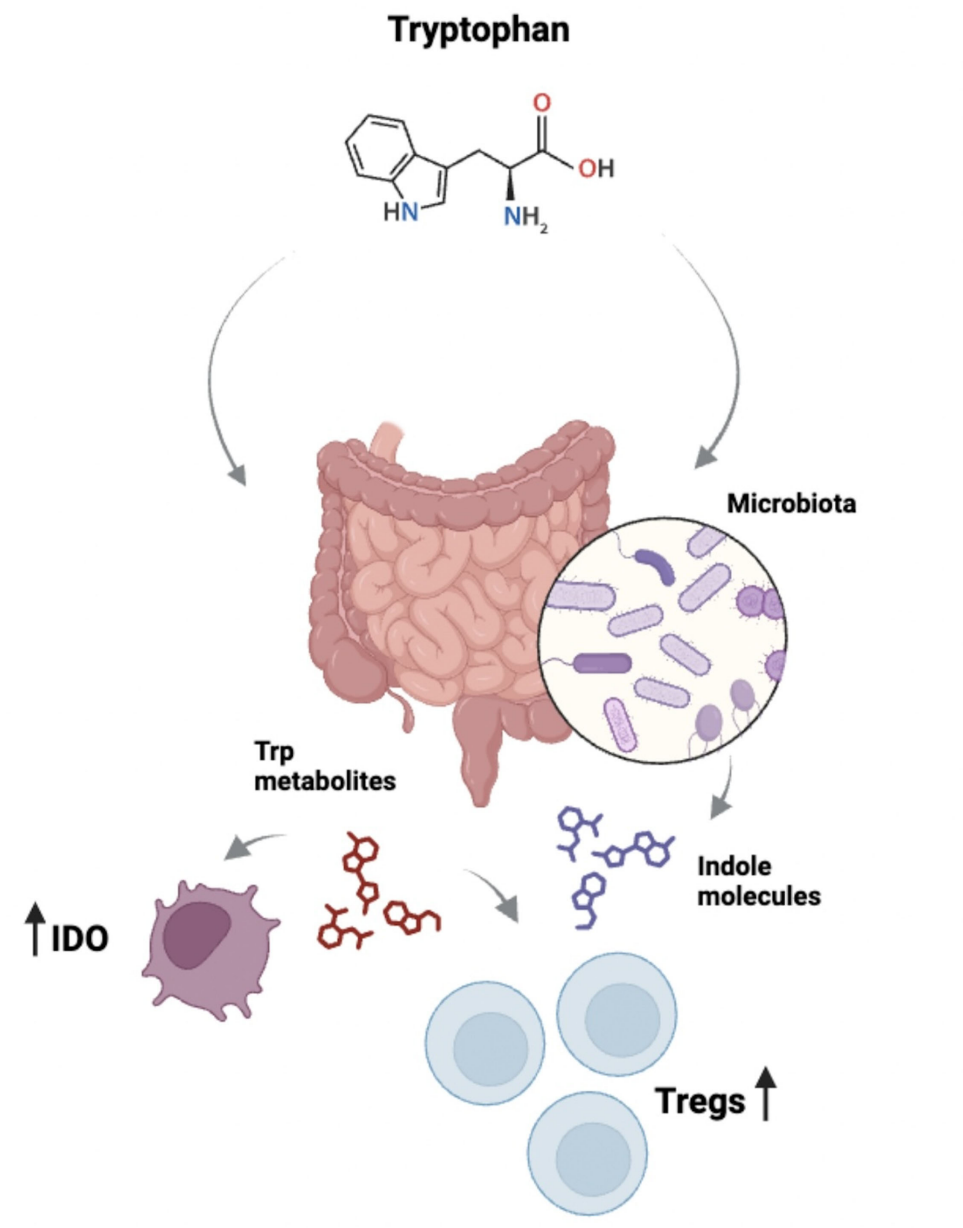
Relationship between Trp and intestinal immunity. Trp is metabolized in the intestine by different cell types, including leukocytes. For example, APCs express IDO which catabolizes Trp and generates metabolites such as kynurenines, which can be up taken by other cells, affecting immune responses. Of relevance, kynurenines modulate the pro-inflammatory/anti-inflammatory balance by impacting the function of Th17 and Tregs. Importantly, the intestinal microbiota also plays a role via its capacity to metabolize Trp, which results in the generation of molecules with immune regulatory properties.

In mice, this AhR/IL-22 axis has also been implicated with microbiota since *Lactobacilli* are shown to expand and produce AhR ligands that regulate *Il22* transcription in intestinal immune cells (38). Besides, few years ago the production of microbiota-derived Trp metabolites were demonstrated to inhibit the immune response against LPS in the intestine, decreasing the secretion of IL-6 and regulating the metabolism of fatty acids (43). A general view on how Trp metabolism impacts the intestine is depicted in **Figure 2**.

## Tryptophan metabolism: implications in transplantation

### Hematopoietic transplantation

A recent study indicates that murine fecal microbiota transplant into aged mice promotes the abundance of *Lachnospiraceae*, and that Trp-associated metabolites recovered hematopoiesis and rejuvenated aged hematopoietic stem cells (44). Other studies have indicated that decreased production of AhR ligands such as microbiota-derived indoles and Trp derivatives can induct allogeneic T cell reactivity after allogeneic hematopoietic stem cell transplantation (HSCT) (45) and that plasma amino acids (including Trp) from HSCT patients are reduced in those experiencing sinosoidal obstruction syndrome and Graft-versus-Host Disease (GvHD) (46). On these patients, low levels of plasma amino acids correlated with high amounts of C reactive protein and IL-6, indicating a relationship between decreased plasma amino acids and a pro-inflammatory condition. However, other studies demonstrated increased KYN in plasma from HSCT patients (47), and increased intestinal IDO expression in addition to the Trp metabolites QUIN and KYN in urine that correlate with GvHD severity (48). These results indicate that Trp is involved in the response to HSCT, and that the specific metabolite evaluated, either the amino acid itself (Trp) or an intermediary such as KYN, QUIN or other, has to be carefully considered in order to conclude its positive or negative association with the response to transplantation.

### Solid organ transplantation

Several reports have been published suggesting a relationship between Trp metabolism intermediaries and diminished allograft rejection. Nearly twenty years ago, it was demonstrated that subcutaneous administration of a mixture of KYN and 3-hydroxyanthranilic acid (3-HAA) resulted in prolonged skin allograft survival in rats (49). Besides, administration of 3-HAA plus allogeneic DCs improved rat heart allograft survival, which was associated with depletion of host T cells (50).

Using a cornea transplantation model, it was demonstrated that IDO is expressed during allograft rejection and its overexpression prolonged allograft survival. The authors showed that systemic administration of the Trp metabolite 3-hydroxykynurenine (3-HK) extended corneal allograft survival, which correlated with a reduction of circulating leukocytes (51). Lassiter et al. used a different strategy, namely administering nanoparticles that had been previously shown to induce the expression of IDO, thus incrementing the production of Trp metabolites such as 3-HK and 3-HAA. Intraperitoneal injection (i.p) of these nanoparticles into skin-transplanted mice attenuated graft rejection. Similar results were obtained when the animals were directly injected with 3HK. Additionally, the authors pretreated pig kidneys with the nanoparticles before transplantation and showed that rejection was reduced whereas the expression of Trp metabolism-related enzymes was upregulated (52). Other research has investigated the Trp pathway indirectly by studying the activation of AhR. In a skin transplant model, i.p administration of two different AhR agonists, 2,3,7,8-tetrachlorodibenzo-*p*-dioxin (TCDD) and FICZ, resulted in opposite outcomes: TCDD prolonged allograft survival along with an increment in splenic Tregs via IDO up-regulation, whereas FICZ accelerated transplant rejection and increased IL-17 production. The authors also showed a possible crosstalk between the activation of AhR signaling and the expression of IDO (53). Similarly, i.p administration of the AhR agonist N-(3,4-dimethoxycinnamonyl) anthranilic acid (3,4-DAA) into liver-transplanted rats resulted in prolonged allograft survival which correlated with an increase in Tregs and in PD-1 expression on T cells, whereas administration of the AhR inhibitor CH223191 had the opposite effect (54). These discrepancies are attributed to the characteristics of the ligands, which vary in the binding properties to AhR (long-lasting versus transient) and their resistance to be metabolized. Thus, TCDD´s immunosuppressive function may rely on the sustained AhR activation and its resistance to be metabolized, whereas FICZ´s proinflammatory effects may be linked to its transient AhR signaling and rapid metabolism. Beside these facts, the dose and the targeted cells are also crucial on AhR activation by these ligands (55, 56).

## Microbiota-derived metabolites involvement in transplantation

### Hematopoietic transplantation

The contribution of the microbiota to transplant outcome is now recognized. It has been reported that pre-transplantation irradiation and chemotherapy cause dysbiosis, affecting the levels of endogenous indoles. Thus, mice colonized with TnaA-expressing *E. coli* prior to bone marrow transplantation (BMT) were protected from weight loss, indole reduction and bacterial translocation into mesenteric lymph nodes, and showed extended survival from reduced GvHD. Similarly, BMT mice gavaged with indole-3-carboxaldehyde (ICA, a commensal bacterial product) also showed weight gain, improved survival, and generation of CD4+ and CD8+ T cell-mediated immune tolerance. Finally, the authors demonstrated that ICA-treated mice displayed a type-1 IFN gene signature in gut epithelium previously associated with intestinal homeostasis and repair. Accordingly, ICA treatment failed to protect IFNα-/- mice from weight loss or death upon total body irradiation, demonstrating a type-I IFN-dependent relationship between commensal bacterial indoles and the damaged associated to conditioning pre-transplantation (57).

Alterations in metabolites from gut-derived bacteria have also shown to condition acute GvHD in patients receiving allogeneic hematopoietic stem cell transplantation (58). Due to this, an ongoing Phase II clinical trial is evaluating the effect of oral administration of resistant potato starch (RPS) as prebiotic in hematopoietic transplant patients (59). The administration of RPS increased butyrate levels in feces and modified the concentration of several metabolites in plasma, including L-KYN. However, it is still unknown the potential clinical benefits of RPS administration (59, 60). The above correlates with microbiota 16S sequencing of stools from hematopoietic transplantation patients, where higher indole and butyrate levels associated with a higher gut-bacterial diversity. However, this study does not conclude a direct link between gut-bacterial diversity and overall survival after transplantation (61).

### Solid organ transplantation

Although remaining to be tested, it is conceivable that the indole pathway may also affect alloimmunity in solid organ transplantation, as the microbiota is now a well-established modulator of transplant outcomes (62–64). In the clinical setting, Trp metabolism has been investigated in transplant patients. Early studies focused on indoxyl sulfate (IS), a microbiota-derived Trp metabolite considered as a uremic toxin, described its levels in plasma from chronic kidney disease (CKD) and kidney transplantation patients. The results demonstrated that IS levels are elevated in plasma of CKD patients but decreased after receiving a kidney transplant, suggesting an association between peripheral IS and kidney health status (65–67). More recent, a study followed plasma levels of IAA (also considered a uremic toxin and a AhR ligand as IS) in CKD patients. IAA levels were high in CKD patients, and decreased after receiving a kidney transplant, implying a potential correlation between the concentration of this Trp metabolite and kidney health; however, the potential use of IAA as predictor factor might be only applicable to non-transplanted patients since elevated levels of IAA correlated with cardiac events and mortality on these group and not in those receiving a transplant (68).

Furthermore, a metabolomic analysis in urine from kidney transplant individuals showed the presence of KA and tryptamine in a group of patients that had stopped taking immunosuppressive medications and had developed spontaneous operational tolerance (SOT). The authors associated this finding with their previous identification in SOT patients of increased *Proteobacteria*, a phylum of Gram-negative bacteria that includes species known for producing AhR ligands (69). Other report by DeLa Cruz et al. reported the fecal microbiota landscape and metabolites from heart-transplanted (HT) patients. Peri-transplant fecal samples from HT patients displayed reduced diversity of commensal anaerobic bacteria within the phylum *Firmicutes* when compared with healthy controls, correlating with decreased levels of short chain fatty acids (SCFAs), such as fecal butyrate, acetate and propionate, and secondary bile acids (70). Inasmuch as these metabolites may contribute to AhR signaling (71, 72), it is tempting to speculate that AhR signaling is altered in transplanted patients, a dysregulation that may lead to an inflammatory state.

## Discussion

As presented here, Trp and its metabolites play a role on immune regulation in the transplantation context. Although the importance of diet, host inflammatory status, use of antibiotics, and microbiota composition/diversity is being established, we believe that dissecting the role of Trp and specific metabolites and pathways are required. On this point, we highlight the demonstrated interest on KYN and indoles over KA and AhR signaling. Since data from animal models is not always representative of the human scenario, further human microbiota studies are required to define its role in the response to an allograft, in both hematopoietic stem cells and solid transplantation. This new knowledge may allow for the identification of new therapeutic targets to prevent allograft rejection.
